# Changes in telomere length and senescence markers during human ovarian tissue cryopreservation

**DOI:** 10.1038/s41598-021-81973-3

**Published:** 2021-01-26

**Authors:** Boram Kim, Ki-Jin Ryu, Sanghoon Lee, Tak Kim

**Affiliations:** Department of Obstetrics and Gynecology, Korea University College of Medicine, 73, Inchon-ro, Seongbuk-gu, Seoul, 02841 South Korea

**Keywords:** Cell biology, Medical research

## Abstract

Ovarian tissue cryopreservation is considered as a useful option to preserve fertility for cancer patients. This study purposed to evaluate the change of telomere length and senescence markers during human ovarian tissue cryopreservation. Ovarian tissues were obtained from women who underwent benign ovarian surgery in the gynecology research unit of a university hospital with prior consent and IRB approval. DNA was extracted from the ovarian tissues using a DNeasy tissue kit and telomere lengths in the DNA samples were determined by real time PCR before and after cryopreservation. All tissues were stained with hematoxylin–eosin and subjected to immunohistochemistry and TUNEL assays. Other senescence markers, including p53, p16, p21, and phospho-pRb proteins, were evaluated using western blot analysis. Ovarian tissues were collected from ten patients and prepared for slow freezing with the same size of diameter 4 mm and 1 mm thickness. Mean age of patients was 26.7 ± 6.2 years (range, 16–34 years), and ovarian tissues were cryopreserved and thawed 4 weeks after cryopreservation. The mean telomere length was significantly decreased after cryopreservation (9.57 ± 1.47 bp vs. 8.34 ± 1.83 bp, *p* = 0.001). Western blot analysis revealed that p53, p16, and p21 proteins increased and phospho-pRb protein expression decreased after ovarian tissue cryopreservation. Ovarian tissue cryopreservation and transplantation is regarded as one of promising options for fertility preservation. However, clinicians and researchers should be aware of possible irreversible DNA changes such as shortening of telomere length and alterations of other senescence markers in human ovarian tissues.

## Introduction

The life expectancy of female cancer survivors of reproductive age has increased, and the various treatments to preserve their fertility and quality of life have received considerable attention worldwide^1,2^. Recent guidelines for fertility preservation have emphasized that health care providers should provide fertility preservation consultations to patients diagnosed with cancer as early as possible^3,4^. Although oocyte and embryo cryopreservation are still considered standard practice, ovarian tissue cryopreservation has advanced quickly and is no longer considered experimental. In fact, it is expected to be accepted as standard therapy in the near future^3^. There are remarkable benefits that stem from ovarian tissue cryopreservation, including availability for prepubertal girls, restoration of reproductive function, and a lack for the need for male partners and the need to delay the ovarian stimulation or oocyte harvest process^5^. A recent prospective cohort study showed that ovarian tissue cryopreservation followed by transplantation is successful with regard to the live and clinical birth rates, although there was only a small number of patients returning to use their cryopreserved gametes^6^. The study showed high efficacy at fertility preservation, allowing for natural conception in almost half of patients and restoring ovarian function in 93.2% of patients in that study. To confirm the safety of this method, however, more data should be gathered about the irreversible changes that occur in ovarian cells after cryopreservation, such as proliferation disability, DNA damage, and acceleration of cellular senescence^7^.

The telomere is a specialized structure that consists of proteins and nucleotides of TTAGGG repeats at the ends of eukaryotic chromosomes. Telomeres are essential for genome stability and the regulation of cell proliferation^8^. A telomere’s length gradually shortens with every cell division^9^. The total number of cell divisions cannot exceed the so-called Hayflick limit; at this point, the telomeres reach a critical length, meaning that the cell has become senescent^10,11^. This telomere shortening theory and accumulated experimental data suggest that telomeres are a promising marker of cellular senescence^8^. A few experimental studies have reported that cryopreservation induces telomere shortening and cellular senescence in human stem cells and retinal pigment epithelial cells^12,13^.

Cell senescence is induced by multiple stimuli and various stressors can cause telomere shortening and dysfunction or DNA damage. DNA damage responses and senescence signals change the cellular phenotype through the activation of the ARF-p53-p21 pathway, which is a partially telomere-dependent pathway, and the p16-pRB pathway, which is independent of telomere dysfunction^14,15^. There is reciprocal regulation between the p53-p21 and p16-pRB pathways. The consequence is activation and regulation of these senescent pathways, which may play a central role in ovarian tissue damage or repair during the cryopreservation and thawing process.

To the best of our knowledge, no previous study investigated the effect of the cryopreservation of human ovarian tissue on cellular senescence by measuring telomere length or changes in other senescence markers. This experimental study evaluated telomere length and senescence marker changes after human ovarian tissue cryopreservation using a slow-freezing method. We also studied immunohistopathological findings and terminal deoxynucleotidyl transferase dUTP nick-end labeling (TUNEL) assays to assess DNA damage induced by cryopreservation.

## Materials and methods

### Tissue samples

Human ovarian cortex tissue was obtained from 10 patients who underwent benign ovarian surgery. All patients provided informed consent. If subjects were under 18 years of age, consent was obtained from a parent and/or legal guardian. No patients underwent preoperative chemotherapy or radiotherapy. The ovarian tissues were punctured using a biopsy punch (Kai Industries C., Ltd., Gifu, Japan) to create samples with a 4-mm diameter and 1-mm thickness. This study was conducted in accordance with the Declaration of Helsinki with the approval from the Institutional Review Board of Korea University Anam Hospital (no. ED11138).

### Slow-freezing protocol

**Figure 1:**
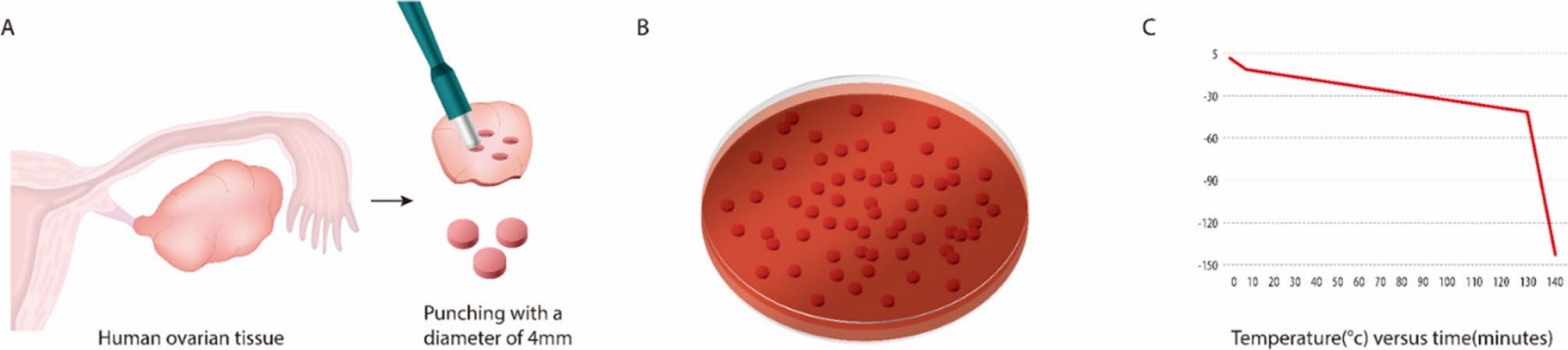
Ovarian tissue sampling and slow-freezing process. (**A**) Pictorial representation of the creation of the 4-mm-diameter ovarian tissue samples. (**B**) Fragments of human ovarian tissue cut to a small size (4 mm). (**C**) The small tissue samples were split into tubes and frozen at the same rate shown here. The graph shows the temperature dropping over time using a programmable refrigerator. The tissue was slowly frozen from – 7° to – 40° and rapidly frozen from – 40° to – 130°. After freezing, the tissue was stored in liquid nitrogen.

The slow-freezing and thawing of sample tissues in this study followed the protocol described in our previous study^16^. The ovarian tissue sections were transferred to a basic solution containing 5% serum replacement supplements (SSS; catalog 99193, Irvine Scientific, CA, USA) in M199 culture medium (catalog M4530, Sigma Aldrich, MO, USA). The cryoprotectant was added in three successive dilutions. Five percent SSS–supplemented tissue sections in M199 culture medium were exposed to 7.5% dimethyl sulfoxide (DMSO; catalog D2650, Sigma Aldrich, UK) for 5 min, treated with 10% DMSO for 15 min, and treated with 12.5% DMSO for 15 min. All procedures were performed on ice. Next, the ovarian tissue sections were transferred into a cryotube containing 1 mL of final freezing media containing 12.5% DMSO and 5% SSS in M199 culture medium. The cryotubes were cooled in a programmable controlled-rate freezing device (Planer PLC, UK) with a slow-freezing protocol as described previously^16^ : cooling from 4 to − 7.0 °C at − 2.0 °C/min, followed by manual seeding and cooling to − 40.0 °C at − 0.3 °C/min and − 140 °C at − 10 °C/min. After freezing, the samples were ultimately stored at − 196 °C in liquid nitrogen (Fig. 1).

### Thawing protocol

The cryotube vials, which were stored for 4 weeks, were transferred from liquid nitrogen to a shaking water bath at 37 °C. When the vial was fully thawed, half of the supernatant was removed and replaced with the same volume of wash solution containing 5% DMSO in the primary medium. The vials were incubated at room temperature for 10 min. After incubation, half of the supernatant was again removed and replaced with an equal volume of wash solution. The vial was then incubated at room temperature for 5 min.

### Telomere length measurement

DNA was isolated from the patients’ ovarian tissue using a QIAamp DNA mini kit (QIAGEN, Hilden, Germany) according to the manufacturer’s instructions. The tissue samples were incubated with protease K (20 μl) and lysis buffer (180 μl) at 56 °C for 3 h to isolate the DNA. The extracted DNA was sent to a commercial company (MEDIAGE Biological Age Measurement System, MEDIAGE Research Center, Gyeonggi-do, South Korea) for telomere length measurement. Quality control of the DNA (purity and concentration) was performed using a NanoDrop 2000/2000c Spectrophotometer (Thermo Fisher Scientific, DE, USA). Telomere length was determined by real-time quantitative polymerase chain reaction (RT-qPCR) which uses less DNA and requires much less time to perform with comparable accuracy compared to the traditional Southern blot method for measuring terminal restriction fragment lengths^17^. RT-qPCR was performed using a pair of telomere primers, telg, ACACTAAGGTTTGGGTTTGGGTTTGGGTTTGGGTTAGTGT and telc, TGTTAGGTATCCCTATCCCTATCCCTATCCCTATCCCTAACA, using a QuantStudio 6 Flex Real-Time PCR system (Life Technologies Corp., CA, USA). The detailed techniques were followed the methods described in the previous studies^17^.

### Histologic evaluation

The tissues were fixed in 4% paraformaldehyde in phosphate-buffered saline (PBS), embedded in paraffin, and sliced into 3-μm sections. The sections were deparaffinized with using xylene and dehydrated stepwise in graded ethanol solutions (50%, 70%, 80%, 90%, and 100%). The slides were stained with hematoxylin–eosin (H&E) and photographed using a microscope (BX-51; Olympus, Tokyo, Japan). The follicles were classified according to their developmental stages based on the granulosa cell morphology. A primordial follicle was defined as an oocyte surrounded by a single fusiform granule cell, while a primary follicle was defined as an oocyte surrounded by a single layer of cuboidal granulosa cells. Secondary follicles were surrounded by six to eight layers of cuboidal granulosa cells with no visible antrum. To avoid repetition, only follicles containing an oocyte with a visible nucleus were counted. Total number of primordial follicles was counted in each samples of the punched ovarian tissues sized 12.57 mm^2^. Structural integrity of the follicles was assessed using appropriate criteria described in the previous studies^18^. Morphologically intact follicles were identified based on the oocyte integrity by two examiners.

### Immunohistochemistry

IHC staining with γ-H2AX was performed to evaluate double-strand DNA breaks in cryopreserved human ovarian cells. Unstained and paraffin-embedded ovarian tissue slides were deparaffinized with xylene and rehydrated with graded ethanol. The slides were left in the target extraction solution (heat-induced epitope retrieval, Citrate Buffer, pH 6.0; Sigma Aldrich, MO, USA) for 15 min in a microwave for antigenic retrieval. The samples were blocked with 3% peroxide blocking solution for 5 min and washed with 1 × Tris-buffered saline with Tween-20 buffer. After blocking, the sections were incubated with γ-H2AX antibody dilution solution (1:500 dilution; Bethyl, Montgomery, USA) for 1 h at room temperature. The slides were incubated for 60 min with antibody in the Polink-2 Plus HRP Broad Kit with DAB (D41-18; GBI Labs, Bothell, WA, USA). The sections were treated with a liquid 3,3′-diaminobenzidine tetrahydrochloride substrate (D41-18; GBI Labs, Bothell, WA, USA) and counterstained with Mayer’s hematoxylin (HMM125; Scytek, Logan, UT, USA). The slides were dehydrated, mounted, and subjected to microscopic examination. Those processes were replicated three times for each sample. The human colon cancer tissues were used as positive control for γ-H2AX.

### TUNEL assay

The DNA fragmentation was analyzed using the TUNEL assay. After deparaffinization, the samples were rehydrated with graded ethanol and immersed in 4% formaldehyde in PBS (AB216603, Hyclone) for 15 min. The samples were processed using the Click-iT Plus TUNEL Assay for In Situ Apoptosis Detection, Alexa Fluor 488 dye (C10617; Thermo Fisher). The nuclei were stained and mounted with VECTASHIELD mounting medium (H-1400; Vector Laboratories, CA, USA). TUNEL-positive cells produced green fluorescence. Immunofluorescence images were obtained using a fluorescence microscope (Olympus). Those processes were replicated three times for each sample.

### Western blot analysis

The tissues were lysed in 1× radioimmunoprecipitation assay buffer (BRI-9001; BioPrince, Gangwon, South Korea) supplemented with Complete Protease Inhibitor (04693159001; Roche, Basel, Switzerland) and Phosphatase Inhibitor (04906837001; Roche) for 30 min at 4 °C. The lysates were centrifuged at 13,000 rpm for 30 min at 4 °C. After centrifugation, the protein concentration was measured according to the Bradford assay method (Bio-Rad, Hercules, CA, USA). The resulting supernatants (45 µg of protein) were subjected to sodium dodecyl sulfate polyacrylamide gel electrophoresis using 12% gels.

The primary antibodies pRb (1:1000; D20B12; Cell Signaling, Danvers, MA, USA), p16 (1:1000; D3W8G; Cell Signaling, Danvers, MA, USA), p21 (1:1000; 12D1; Cell Signaling, Danvers, MA, USA), p53 (1:1000; sx-126; Santa Cruz Biotechnology, Dallas, TX, USA), and anti-β-actin (1:1000, sc-47778, Santa Cruz Biotechnology, Dallas, TX, USA) were incubated with the blots at 4 °C overnight with gentle shaking. Thereafter, they were incubated with a goat anti-rabbit secondary antibody (1:5000; ab6721; Abcam, Cambridge, UK) at room temperature for 60 min. Immunoreactive proteins were visualized using SuperSignal West Pico plus Chemiluminescent Substrate (QI225261; Thermo Fisher, Waltham, MA, USA) and detected on a Medical X-ray Blue film (Agfa-Gevaert, Mortsel, Belgium) and using a chemiluminescence system (ChemiDoc Touch Imaging System; Bio-Rad, Hercules, CA, USA). Those images were analyzed using an ImageJ program and Image Lab Software (Bio-Rad, CA, USA).

### Statistical analysis

The results from the IHC staining and TUNEL analyses were quantified using ImageJ. The results of IHC, TUNEL, western blot, and telomere lengths within the samples were compared using Student’s *t*-test or analysis of variance (ANOVA) using SPSS version 12.0 software (SPSS Inc., CA, USA). *P* values < 0.05 were considered statistically significant.

## Results

### Telomere lengths

**Figure 2:**
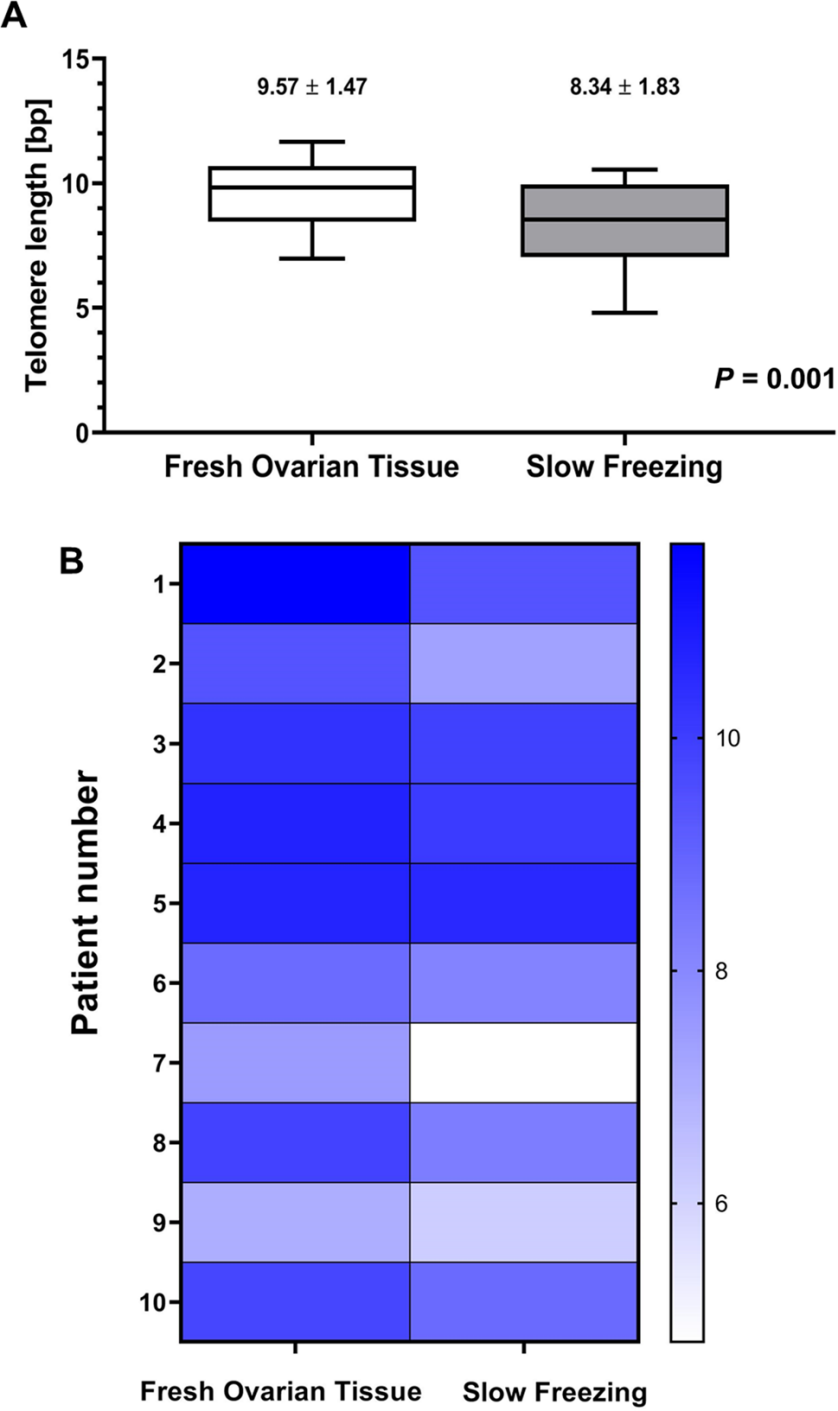
Difference in telomere length between the control and slow-frozen ovarian tissue. (**A**) Comparison of mean telomere length in DNA extracted from the cells of the control and slow-frozen ovarian tissues obtained from ten patients. Mean telomere length was significantly reduced after cryopreservation (*p* = 0.001). (**B**) Individual telomere lengths were compared between the two groups and marked with a heat map.

Mean age of the 10 patients was 27.1 ± 6.0 years (range, 16–34 years), and cryopreserved ovarian tissues were thawed 4 weeks after cryopreservation. Telomere length was evaluated before and after the slow-freezing and thawing process (Table 1). There was a consistent trend of decreasing telomere lengths after cryopreservation in the 10 studied samples; mean telomere length was significantly reduced after cryopreservation (9.57 ± 1.47 bp vs. 8.34 ± 1.83 bp, *p* = 0.001; Fig. 2A). Individual telomere lengths were compared between the two groups and marked with a heat map (Fig. 2B). Telomere length was reduced by a mean 12.8% after cryopreservation compared to fresh ovarian tissues.

**Table 1:** Patient clinical characteristics and comparison of telomere lengths in DNA extracted from the cells of the control and slow-frozen ovarian tissues obtained from ten patients.

Patient no	Age	Telomere length (bp)		Diagnosis		Treatment
		Control	Slow-freezing			
1	21	11.665	9.429	Endometrioma, right ovary		Pelviscopic right ovarian cystectomy
2	25	9.401	7.329	Endometrioma, both ovaries		Pelviscopic bilateral ovarian cystectomy
3	28	10.310	9.918	Breast cancer		Robot-assisted right oophorectomy for ovarian tissue cryopreservation
4	32	10.739	10.065	Endometrioma, both ovaries		Pelviscopic bilateral ovarian cystectomy
5	34	10.680	10.540	Endometrioma, left ovary		Pelviscopic left oophorectomy
6	16	8.783	8.123	Dermoid cyst, right ovary		Pelviscopic right ovarian cystectomy
7	22	7.473	4.809	Dermoid cyst, right ovary		Pelviscopic right ovarian cystectomy
8	34	9.872	8.30	Endometrioma, right ovary	Robot-assisted right ovarian cystectomy	
9	28	6.970	6.132	Endometrioma, both ovaries	Pelviscopic bilateral ovarian cystectomy	
10	31	9.780	8.800	Dermoid cyst, left ovary	Pelviscopic left ovarian cystectomy

### Histological analysis

**Figure 3:**
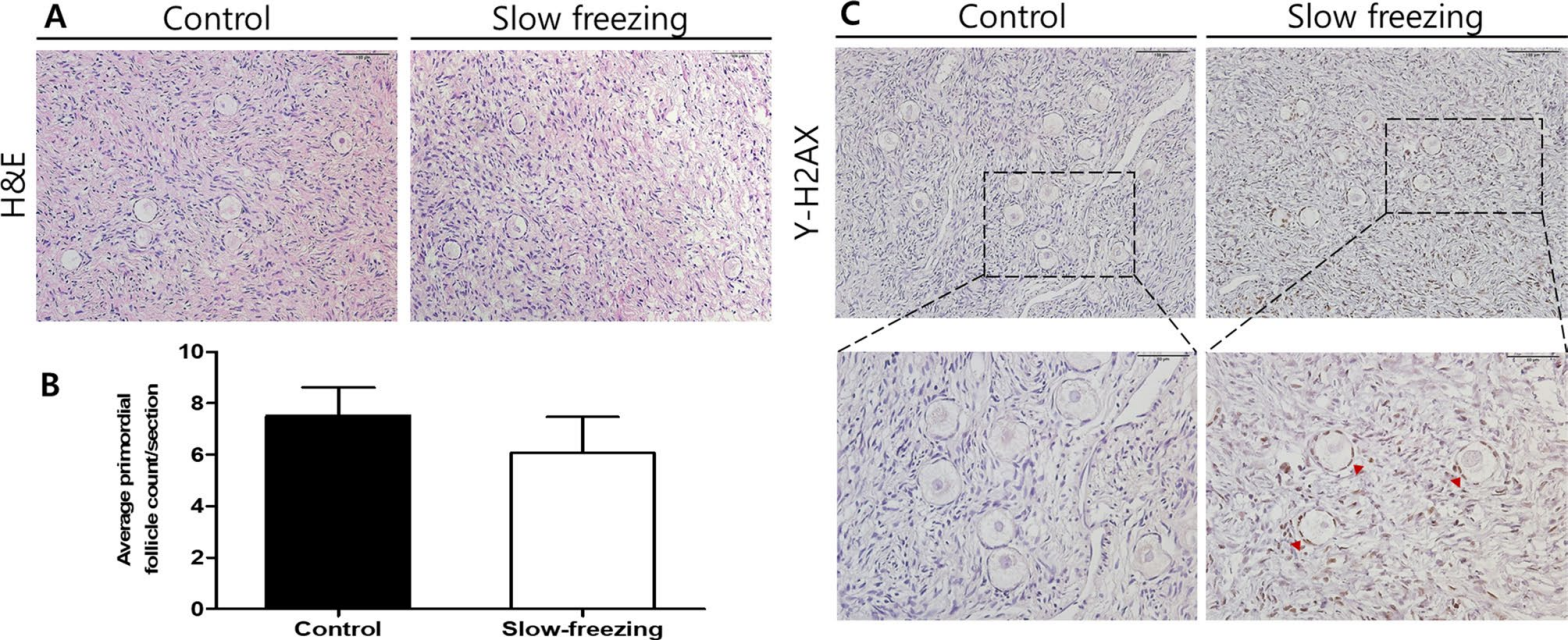
Immunostaining results of human ovarian tissue before and after cryopreservation using the slow-freezing method. (**A**) Hematoxylin (blue) and eosin (red) staining was performed to determine morphological changes and follicle number in human ovarian tissue. No significant morphological changes were detected in either tissue. (**B**) Graph showing the mean number of follicles in the tissue obtained from the patients. (**C**) Histological features of double-strand DNA breaks using γ-H2AX staining. The DNA-damaged nuclei are stained dark brown (arrowheads). Scale line, 100 μm.

A total of ten ovarian tissues were randomly selected for each of 10 fragments, and H&E staining was performed to confirm morphological changes and follicle count. No significant morphological changes to the follicles were seen in the control or slow-frozen tissues (Fig. 3A). The mean number of primordial follicles was higher in the control tissue than in the slow-frozen tissue (7.508 vs. 6.081, *p* = 0.425), (Fig. 3B). Figure 3C shows a representative picture of the immunohistochemistry staining results for γ-H2AX to evaluate double-stranded DNA cleavage in the primordial follicles. No damaged nuclei were found in the control tissue. However, in slow-frozen tissues, while no nuclear damage was observed in the oocyte, the nuclei of the granulosa cells around the follicles were damaged (Fig. 3C). The mean percentage of γ-H2AX-positive cells was significantly higher in the slow-frozen tissues compared to the control tissues (51.029 ± 10.62 vs. 1.667 ± 2.887, respectively, *p* < 0.001).

**Figure 4:**
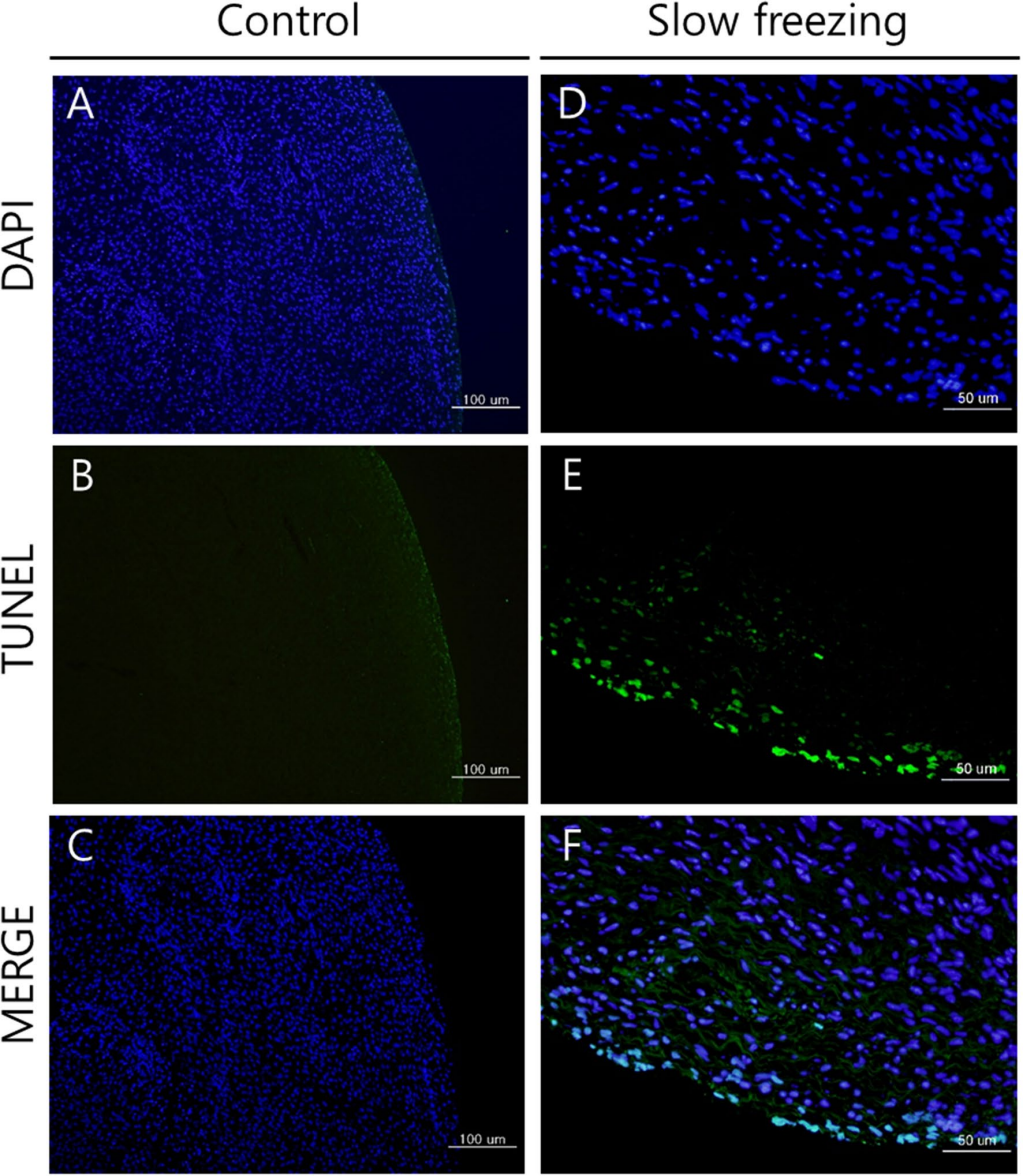
DNA double-strand breaks. The terminal deoxynucleotidyl transferase dUTP nick-end labeling (TUNEL) assay confirmed DNA double-strand breaks. The DNA double-strand breaks are indicated in green, and 4′,6-diamidino-2-phenylindole (DAPI) staining (blue) shows the nuclei. Control [(**A**) TUNEL assay; (**B**) DAPI staining; (**C**) merged, ×100]; slow-freezing [(**D**), TUNEL assay; (**E**) DAPI staining; (**F**) merged, ×200].

The results of the TUNEL analysis of DNA double-strand breaks are shown in Fig. 4. Blue indicates nuclei shown with 4′,6-diamidino-2-phenylindole staining, and green indicates damage to the nuclei. The proportion of TUNEL-positive cells that produced green fluorescence was higher in the slow-frozen tissues than in the control tissues. As in the γ-H2AX staining results shown in Fig. 3, cell nuclear damage occurring around the follicle was also observed in the TUNEL analysis (Fig. 4). The tissue surface shows the degree of tissue damage caused by the freezing of the ovary tissue. The mean percentage of TUNEL-positive area was significantly larger in the slow-frozen tissues compared to the control tissues (0.115 ± 0.089 vs. 0.014 ± 0.010, respectively, *p* = 0.006).

### Analysis of aging-related proteins in ovarian tissue

**Figure 5:**
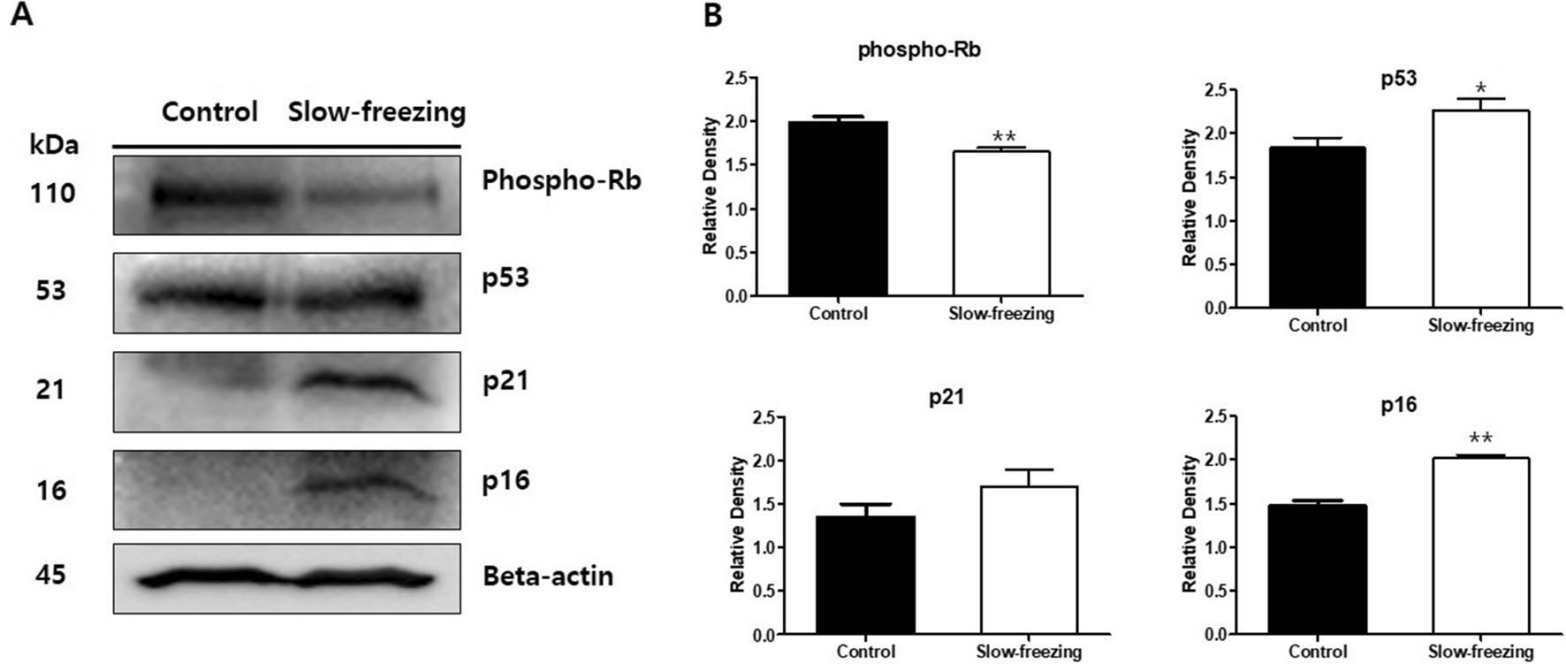
Western blot analysis of phospho-pRb, p53, p21, and p16INK4a in slow-frozen and control ovarian tissues. (**A**) Based on the senescence protein marker p16, four related proteins were measured in the same human ovarian tissue. Parallel blots were probed with β-actin antibodies as a protein quantification control. Full-length blots are presented in Supplementary Figure 1. (**B**) Graphs of the fold-change value from the control after quantification of the western blot analysis results. Images were acquired with the ChemiDoc Imaging Systems (BIO-RAD) and band intensities were quantified with ImageJ software. Paired Student's t-test was used in these analyses. *p-value < 0.05. **p-value < 0.005.

A western blot analysis was performed to determine the aging progress of ovarian tissue after freezing using senescence protein markers. Western blot analysis showed that p53, p16, and p21 protein levels increased and phospho-pRb levels decreased after ovarian tissue cryopreservation (Fig. 5). The level of phospho-pRb protein was strongly detected in the control tissue and relatively weakly detected in the slow-frozen ovarian tissue. In contrast, protein levels of p53, p21, and p16 were higher in the frozen tissues than in the control tissues. Full-length blots are presented in Supplementary Figure 1.

## Discussion

The results of this study show that human ovarian tissue cryopreservation using a slow-freezing technique is associated with significant telomere shortening and altered senescence markers, which may indicate accelerated cellular senescence. Immunostaining findings and mitochondrial structures were also changed, indicating that an aging process or DNA damage occurred after cryopreservation.

Telomere length shortening is considered a validated marker of cellular senescence^8^. Only a few studies have investigated the effect of cryopreservation on changes in telomere length and other senescence markers in frozen and thawed cells. Honda et al. reported that slow-frozen and thawed retinal pigment epithelial cells showed accelerated telomere shortening, impaired proliferation, and excessive single-strand DNA breaks compared with non-cryopreserved cells^13^. Based on these findings, the authors suggested that the cryopreservation process induced cellular senescence. Another study showed that the cryopreservation of cancer stem cells induced an increase in senescence-associated β-galactosidase, indicating the acceleration of cellular senescence^19^. To the best of our knowledge, this is the first experimental study to investigate ovarian cryodamage that occurs during human ovarian tissue cryopreservation using senescence markers.

Some types of DNA damage as well as cell division and the senescence process also induce telomere shortening in cells^20,21^. The cellular response mechanism to telomere shortening is similar to the common cellular response to DNA double-strand breaks^8^. DNA damage responses and senescence signals induce cellular phenotype changes and arrest cell proliferation by upregulating the p53-p21 partially telomere-dependent pathway or the p16-pRb pathway, which is independent of telomere dysfunction^14^. The activation of p16 downregulates cyclin D1, while the activation of p21 suppresses cyclin E expression; these phenomena ultimately result in transient cell cycle arrest. These proteins are also called tumor suppressor markers because they play a role in preventing uncontrolled cell replication by inducing replication arrest and apoptosis of tumor cells in response to oncogenic stresses or DNA damage^22^. In line with these findings, the results of the present study indicate that telomere shortening observed in ovarian cells after cryopreservation and thawing is associated with DNA double-strand breaks confirmed by the TUNEL assays and immunostaining histological findings. These changes were also associated with increases in p53, p21, and p16 protein levels and a decrease in phospho-pRb levels in the western blot analysis. Accumulated data from previous studies indicate that cryopreservation of gamete cells or embryos is associated with DNA damage, which could be induced by numerous factors during the cryopreservation process such as cryoprotectant agent toxicity, osmotic shock, or reactive oxygen species^23^, although the underlying mechanisms have yet to be elucidated.

Direct DNA damage, decreased DNA repair system activity, oxidative stress, and inappropriate freezing conditions such as low concentrations of cryoprotectant agents were suggested by one research group as possible mechanisms underlying the association between cryopreservation and telomere shortening^13^. However, an increase in reactive oxygen species, a marker of oxidative stress, was not directly correlated with cryopreservation in their study. Another study reported that sample groups of human adipose-derived mesenchymal stem cells subjected to slow-freezing with various concentrations of cryoprotectant agents, DMSO and fetal bovine serum, showed no differences in telomere length and tumor suppressor marker levels, including p53, p21, p16, and pRb^24^.

Reliable human data on long-term outcomes of ovarian tissue cryopreservation and transplantation are lacking. A previous investigation of the first nonhuman primate derived from an oocyte in ovarian tissue that underwent autologous transplantation showed that the process neither compromised reproductive function nor presented premature cellular aging, as suggested by shortened telomeres^25^. In contrast, the telomeres were longer than those of control monkeys. The authors suggested that a DNA double-strand break or hypoxic damage might be the reason for telomere lengthening, but these mechanisms lack sufficient evidence^26^. These results cannot be compared with ours because the ovarian tissues used in that study were not subjected to the freezing process. Further studies are needed to reveal the long-term outcomes of births after ovarian tissue cryopreservation and transplantation.

Ischemic injury that occurs during graft revascularization during the first few days after transplantation is considered a major contributor to ovarian follicle depletion and the poor results of cryopreservation and transplantation^27^. Conversely, some researchers recently suggested that depletion of the ovarian reserve after fresh or frozen ovarian tissue transplantation might be due to a burst of primordial follicle recruitment after transplantation rather than the ischemic damage itself^28,29^. This idea is supported by the rapid increase in anti-Müllerian hormone (AMH) after the decrease of follicle-stimulating hormone with a subsequent AMH decrease after ovarian tissue transplantation, as shown in previous investigations^28^. Reduced cellular proliferation rates after cryopreservation have also been reported, but these changes appear transient^30^. However, the present study revealed telomere shortening and DNA damage after cryopreservation of human ovarian tissue. These results indicate that irreversible cellular changes occurred during the freezing process before transplantation and follicular recruitment.

Our study is limited because a relatively small number of patients were included in this preliminary study, and the effects of transplantation were not evaluated. However, more than one hundred pieces of human ovarian tissues from the ten patients were assessed to compare the telomere length before and after cryopreservation. Although ischemic ovarian reserve depletion might occur after the transplantation of ovarian tissues^27^, the authors believed that DNA damage during the freezing process is an important finding that affects the outcomes of cryopreservation as well. Second, telomere length was measured in whole fragments of ovarian tissue and not in specific cell types, and data on telomerase activity was not evaluated in this study. However, cellular specific telomere length and telomerase activity have been reported in in vitro fertilization or human production studies^31,32^. To the best of our knowledge, as mentioned above, this is the first experimental study to investigate the telomere length and alterations of other senescence markers focusing on human ovarian tissue during ovarian tissue cryopreservation.

In conclusion, cryopreservation of human ovarian tissue using a slow-freezing technique is associated with telomere length shortening and altered senescence pathway markers. Based on the immunohistochemistry and TUNEL assay results, our findings suggest this is the result of DNA damage that occurred during the slow-freezing and thawing process rather than from the acceleration of cellular senescence. Altogether, these findings suggest that clinicians and researchers should be aware of the possibility of irreversible DNA changes occurring during the cryopreservation of human ovarian tissues. Further evaluation of a large number of patients will be required to confirm the results of this study.

## Supplementary Information

Supplementary Information.
