# Supplementary material for: Changes in telomere length and senescence markers during human ovarian tissue cryopreservation
Source: Sci Rep. 2021 Jan 26;11:2238. doi: 10.1038/s41598-021-81973-3 (PMC7838193; doi:10.1038/s41598-021-81973-3)
Supplement: Supplementary file 1 — Supplementary Information. [file 41598_2021_81973_MOESM1_ESM.pdf]

Changes in telomere length and senescence markers during human ovarian tissue cryopreservation

Running title: Ovarian cryopreservation and telomere length

Boram Kim[¶], Ki-Jin Ryu[¶], Sanghoon Lee[*], Tak Kim

Department of Obstetrics and Gynecology, Korea University College of Medicine, 73, Inchon-ro, Seongbuk-gu, Seoul 02841, Korea

[¶]Boram Kim and Ki-Jin Ryu contributed equally to this work and are thus considered co-first authors.

[*]Corresponding author: Sanghoon Lee, MD, PhD

Department of Obstetrics and Gynecology, Korea University College of Medicine, 73, Inchon-ro, Seongbuk-gu, Seoul 02841, Korea.

Tel: +82-2-920-5310; Fax: +82-2-921-5357; E-mail: mdleesh@gmail.com

Supplementary Material

Supplementary Figure 1. Full-length pictures of the blots

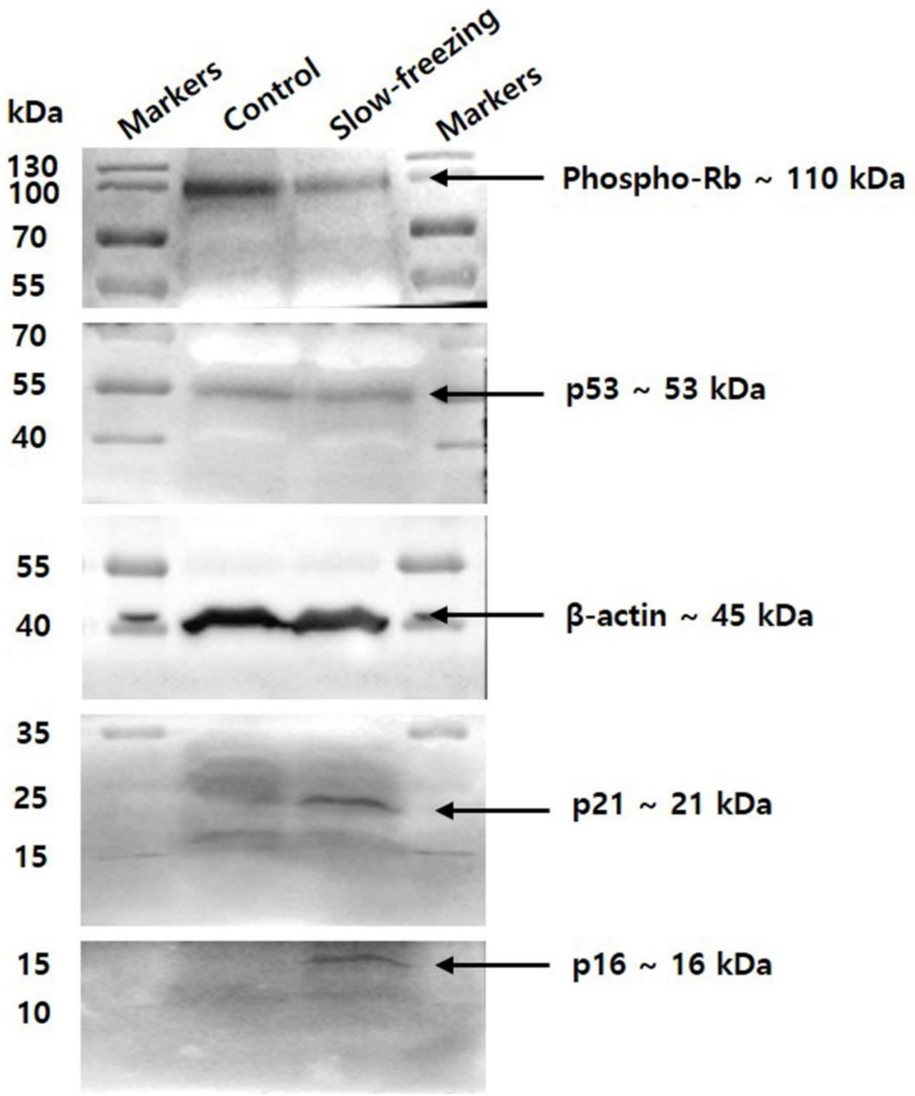

Full-length western blotting images of Figure 5A based on the four senescence protein markers, including phospho-pRb, p53, p16, and p21 in the same human ovarian tissue.
